# Bio-inspired branched Au–Cu nanoalloys for achieving synergistic dual-pathway peroxidase-like activity

**DOI:** 10.1039/d6ra00153j

**Published:** 2026-04-24

**Authors:** S. Priya, S. Sruthi, P. Aiswarya, R. Manu

**Affiliations:** a NSS College Ottapalam Kerala India priyaasudhesh@gmail.com; b NSS College Nemmara Kerala India; c SVNSS College Kerala India

## Abstract

In this study, we report a simple and green one-pot co-reduction strategy for the synthesis of bimetallic Au–Cu branched nanostructures under aqueous conditions. Curcumin was employed as both a reducing and stabilizing agent, eliminating the need for additional chemical reagents. The resulting Au–Cu nanoalloy exhibited a distinctive branched nanotubular morphology and demonstrated excellent peroxidase-like catalytic activity. The catalytic performance was evaluated using the oxidation of 3,3′,5,5′-tetramethylbenzidine (TMB), where the initially colourless substrate was converted into a blue-colored oxidized product. The system exhibited a detection limit of 8 µM for H_2_O_2_. Furthermore, steady-state kinetic parameters, including the Michaelis–Menten constant (*K*_m_) and maximum reaction velocity (*V*_max_), were determined by varying the concentrations of substrates (H_2_O_2_ and TMB). The low *K*_m_ value obtained for TMB indicates a strong affinity of the Au–Cu nanoalloy toward the substrate. A comparative analysis with the curcumin-stabilized gold nanoparticles revealed that the incorporation of copper significantly enhances the catalytic efficiency of the nanostructures. The electron transfer mechanism underlying the catalytic process was further validated using cyclic voltammetry and electrochemical impedance spectroscopy. Overall, this work presents an efficient multimodal sensing platform for the sensitive detection of H_2_O_2_.

## Abstract 2

In this study, we report a simple and green one-pot co-reduction strategy for the synthesis of bimetallic Au–Cu branched nanostructures under aqueous conditions.

## Graphical abstract

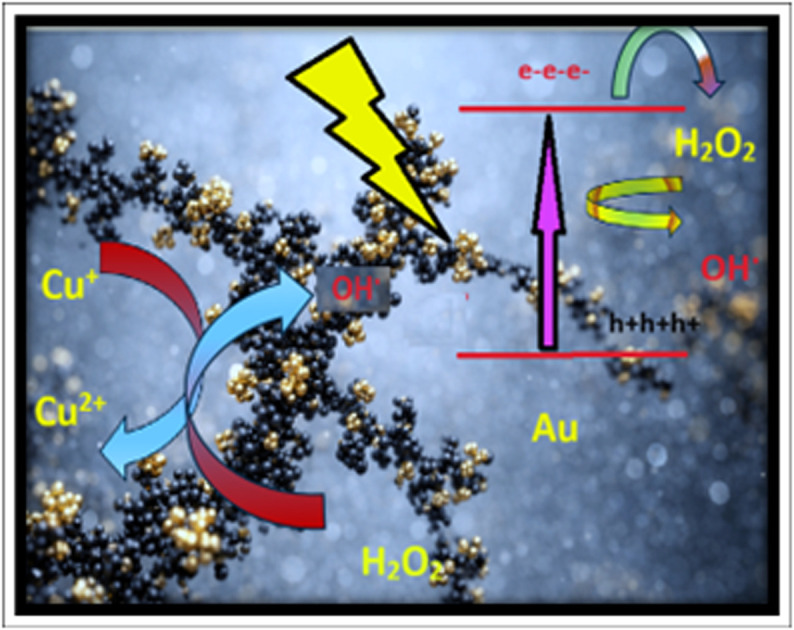

## Introduction

Gold (Au) and copper (Cu) nanoparticles are well-known for their excellent plasmonic and catalytic properties. Gold nanoparticles exhibit size- and shape-dependent tunable optical characteristics, making them highly versatile for applications in medicine, sensing, and optoelectronics. In addition, their chemically stable and biocompatible surface enables facile functionalization for chemical modification and bioconjugation. Copper nanoparticles also display strong plasmonic absorption in the visible and near-infrared regions, which makes them attractive for applications in photonics, sensing, and imaging.^1–7^ However, despite their effectiveness as heterogeneous catalysts, the practical application of Cu nanoparticles is significantly limited by their high susceptibility to oxidation, which leads to deterioration of both their plasmonic and catalytic performance.^8,9^ These limitations can be effectively addressed by alloying Cu with more stable noble metals such as Au or Ag, forming bimetallic nanostructures that exhibit enhanced stability and superior catalytic activity compared to their monometallic counterparts.^10,11^

Nanoalloys typically possess high surface energy and an abundance of active sites, arising from their reduced size, lattice strain, and numerous structural defects. These features collectively contribute to their enhanced catalytic performance. Enzymes, as natural catalysts, exhibit remarkable specificity and play vital roles in diverse fields, such as food processing, pharmaceuticals, and agrochemicals. However, their widespread application is often hindered by limitations such as low operational stability, sensitivity to environmental conditions, and the high costs associated with their production and purification.^12,13^ To address these challenges, artificial enzyme mimics have been developed as robust, cost-effective, and highly stable alternatives to natural enzymes.

Various nanomaterials, including metal nanoparticles, metal oxides, and carbon-based nanostructures, have been reported to exhibit intrinsic enzyme-like activities. These nanomaterials often demonstrate catalytic kinetics comparable to natural enzymes and are therefore referred to as nanozymes.^14,15^ Among the different types of nanozymes, oxidase-, peroxidase-, catalase-, and superoxide dismutase-like systems are the most extensively studied due to their crucial roles in biomedical applications, particularly in regulating reactive oxygen species (ROS).

Briefly, oxidase mimics catalyse the oxidation of substrates by utilizing molecular oxygen as the electron acceptor, resulting in its reduction to water or hydrogen peroxide. Superoxide dismutase (SOD)-like nanozymes facilitate the dismutation of superoxide radicals (O_2_^−^˙) into hydrogen peroxide and oxygen. Catalase-like nanozymes decompose hydrogen peroxide into water and oxygen, thereby mitigating oxidative stress. In contrast, peroxidase-like nanozymes catalyse the oxidation of substrates in the presence of hydrogen peroxide, where the substrate acts as an electron donor.

Peroxidases are a class of natural enzymes capable of catalyzing the oxidation of a wide range of substrates in the presence of hydrogen peroxide and are widely employed in areas such as the food industry, biosensing, and pharmaceuticals.^16,17^ A well-known representative is horseradish peroxidase (HRP), a ferroprotoporphyrin-containing enzyme recognized for its high catalytic efficiency. However, the practical application of HRP is often limited by its susceptibility to harsh environmental conditions, as well as by the high cost and complexity associated with its preparation and purification.^18^

To address these limitations, considerable research efforts have been devoted to the development of peroxidase-mimicking nanomaterials that are stable, cost-effective, reusable, and facile to synthesize. Among various candidates, gold nanoparticles (AuNPs) have attracted significant attention in the biomedical field due to their ease of synthesis, tunable optoelectronic properties, controllable surface functionalization, and excellent catalytic activity arising from their high surface-to-volume ratio. In this context, nanoalloys composed of two or more metallic elements have emerged as promising enzyme substitutes owing to their tunable structure, composition, and physicochemical properties.^19–22^ These materials often exhibit superior catalytic performance compared to their monometallic counterparts, primarily due to synergistic interactions between the constituent elements.

An additional advantage of gold-based nanoalloys lies in their cost-effectiveness, achieved by partially replacing gold with less expensive transition or rare-earth metals while maintaining or even enhancing catalytic efficiency. The catalytic behavior of such nanoalloys is strongly influenced by their composition and structure. For instance, Au–Fe nanoalloys exhibit bifunctional characteristics by combining the plasmonic properties of gold with the magnetic behavior of iron.^23–26^ Similarly, Au–Ag bimetallic nanoalloys have demonstrated tunable peroxidase-like activity under acidic conditions, which can be modulated by varying the alloy composition and solution pH.^27,28^

In the present work, we report a simple one-pot aqueous synthesis of Au–Cu nanoalloys using curcumin as both a reducing and stabilizing agent. A methodological refinement in the synthesis procedure leads to the formation of branched Au–Cu nanoalloy nanostructures. The synthesized materials were comprehensively characterized using SEM-EDX, HRTEM, STEM-EDS, UV-vis spectroscopy, FT-IR spectroscopy, and electrochemical techniques. The Au–Cu nanoalloys exhibited excellent peroxidase-like catalytic activity, as demonstrated by the catalytic oxidation of 3,3′,5,5′-tetramethylbenzidine (TMB) in the presence of H_2_O_2_.

Furthermore, selectivity studies revealed that the nanoalloys possess high specificity for the colorimetric detection of H_2_O_2_, even in the presence of various interfering ions. The sensing mechanism was further elucidated through detailed electrochemical investigations. In addition, the catalytic performance of the Au–Cu nanoalloys was systematically compared with previously reported curcumin-stabilized gold nanoparticles, confirming the enhanced efficiency of the bimetallic system.^29^

## Materials and methods

### Materials

Gold(iii) chloride trihydrate (HAuCl_4_·3H_2_O), copper(ii) sulfate (CuSO_4_), hydrogen peroxide (H_2_O_2_), and dimethyl sulfoxide (DMSO) were purchased from Merck Chemicals, India. Curcumin was extracted from turmeric powder using a standard solvent extraction procedure. Buffer capsules of different pH values were obtained from Alfa Aesar. 3,3′,5,5′-Tetramethylbenzidine (TMB) was also purchased from Alfa Aesar. All aqueous solutions were prepared using distilled water. Sodium carbonate (Na_2_CO_3_) and hydrochloric acid (HCl) were used to adjust the pH of the solutions, and pH indicator paper (range 1–12) was employed to monitor pH.

### Methods

#### Synthesis of the Cu–Au nanoalloy

In a typical synthesis, 2.5 mL of 2 mM HAuCl_4_·3H_2_O and 2.5 mL of 2 mM CuSO_4_ solutions were mixed in a round-bottom flask and heated at 80 °C under constant stirring for 5 min. In a separate step, 0.0046 g of curcumin was dissolved in 20 mL of distilled water, and the pH of the solution was adjusted to 9 using Na_2_CO_3_. The prepared curcumin solution was then added dropwise to the metal precursor mixture under continuous stirring. The reaction was allowed to proceed for 10–15 min at 80 °C, during which a distinct colour change from yellow to light brown was observed, indicating the formation of Au–Cu nanoalloys. The resulting product was collected by centrifugation and washed three times with distilled water to remove any unreacted species. The purified precipitate was subsequently redispersed in 25 mL of distilled water and sonicated for 10 min to obtain a homogeneous brown colloidal solution of Au–Cu nanoalloys. The synthesis protocol was highly reproducible, and the obtained nanoalloy exhibited excellent stability, remaining stable for up to 1 year when stored at 7 °C (TEM images after 1 year are provided in Fig. S1, SI).

#### Detection of peroxidase-mimicking activity of AuNPs

The peroxidase-like catalytic activity of the synthesized Au–Cu nanoalloys was evaluated using 3,3′,5,5′-tetramethylbenzidine (TMB) as a chromogenic substrate. For the assay, 0.0010 g of TMB was first dissolved in 1 mL of dimethyl sulfoxide (DMSO) and subsequently diluted with 9 mL of citric acid buffer (pH 5). The typical reaction mixture consisted of 500 µL of the Au–Cu nanoalloy dispersion, 500 µL of a TMB solution, 200 µL of buffer, and 500 µL of hydrogen peroxide (H_2_O_2_). The reaction was monitored using UV-visible spectroscopy. Upon the addition of H_2_O_2_, a distinct color change from colorless to blue was observed, indicating the oxidation of TMB to its oxidized form (TMB^+^). To optimize catalytic performance, key reaction parameters such as pH and substrate concentration were systematically investigated.

#### Characterisation of the Au–Cu nanoalloy

UV-visible absorption spectra were recorded using a double-beam Thermo Scientific spectrophotometer. The surface functionalization and bonding interactions of the synthesized Au–Cu nanoalloys were analysed by fourier-transform infrared (FTIR) spectroscopy using a Shimadzu FTIR spectrometer. The morphology and structural features of the nanoalloys were examined using high-resolution transmission electron microscopy (HRTEM, JEOL JEM-2100) equipped with a LaB_6_ electron gun, offering a resolution of 0.23 nm. The formation of the alloy was confirmed by scanning transmission electron microscopy coupled with energy-dispersive X-ray spectroscopy (STEM-EDS).

Surface morphology and elemental composition were further investigated using scanning electron microscopy (SEM) coupled with energy-dispersive X-ray spectroscopy (SEM-EDX, JEOL 6390LA/Oxford XMX N), operated at an accelerating voltage range of 0.5–30 kV with a maximum magnification of up to 3 000 00×. Electrochemical characterization was carried out using a Biologic electrochemical workstation in a standard three-electrode configuration, with a glassy carbon electrode as the working electrode, Ag/AgCl as the reference electrode, and a platinum wire as the counter electrode. Electrochemical impedance spectroscopy (EIS) was also performed to evaluate the electron transfer characteristics of the Au–Cu nanoalloys.

#### Enzyme kinetics, interference and real sample analysis

The enzyme-like catalytic behaviour of the synthesized Au–Cu nanoalloys was investigated using the Michaelis–Menten kinetic model. Time-dependent absorbance measurements were carried out to monitor the reaction progress, and the initial rate method was employed to evaluate the effect of substrate concentration on catalytic activity. In this study, hydrogen peroxide (H_2_O_2_) and 3,3′,5,5′-tetramethylbenzidine (TMB) served as substrates. The kinetic parameters, namely the Michaelis–Menten constant (*K*_m_) and the maximum reaction velocity (*V*_max_), were determined by plotting the initial reaction velocity (*V*) as a function of substrate concentration. Further analysis was performed using the Lineweaver–Burk double reciprocal plot, obtained by plotting 1/*V* against 1/[*S*], which enabled more accurate estimation of the kinetic parameters.

Interference studies were carried out using the same peroxidase-like assay in the absence of H_2_O_2_ to evaluate the selectivity of the Au–Cu nanoalloy system in the presence of potential interfering species. Common biomolecules, including glucose, salicylic acid, ascorbic acid, cholesterol, and urea (each at a concentration of 1 mM), were individually tested under identical experimental conditions. The corresponding colorimetric responses were recorded and compared to assess potential interference effects, confirming the selectivity of the nanoalloy system toward H_2_O_2_ detection. For real sample analysis, the developed Au–Cu nanoalloy-based sensing system was applied to detect H_2_O_2_ in milk samples, demonstrating its practical applicability in complex biological matrices.

## Results and discussions

Gold–copper nanoalloys (Au–Cu NAs) were synthesized *via* a simple, green, one-pot co-reduction strategy in aqueous medium, without the use of harsh chemicals. Curcumin functioned as both a reducing and stabilizing agent during the synthesis process, thereby simplifying the reaction system and enhancing its eco-friendly nature. The Au–Cu NAs were prepared using gold and copper precursors in a 1 : 1 molar ratio. Co-reduction is considered an effective approach for nanoalloy synthesis, as it involves the simultaneous reduction of two metal ions with comparable reduction potentials by a common reducing agent. The reaction was carried out under alkaline conditions, during which the solution colour gradually changed from light yellow to dark brown, indicating the successful formation of Au–Cu nanoalloys.

**Fig. 1:**
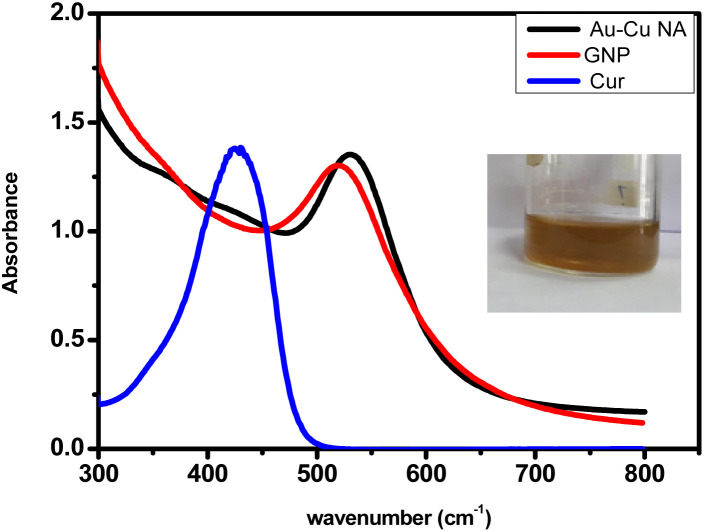
UV-vis spectra of curcumin, GNP and Au–Cu NA. Inset shows the colour of the Au–Cu NA.

As shown in Fig. 1, the UV-visible absorption spectrum of the Au–Cu nanoalloys (NAs) exhibited a distinct surface plasmon resonance (SPR) band. In our previous study, curcumin-stabilized gold nanoparticles (Cur-GNPs) displayed a characteristic SPR peak at 520 nm, whereas the Au–Cu NAs showed a red-shifted SPR peak at approximately 550 nm, indicating an increase in particle size and/or changes in morphology.^29^ The inset of Fig. 1 shows the colour of the synthesized Au–Cu NA. In contrast to the observations reported by Spataro *et al.*,^30^ who observed a dampening of the SPR band during alloy formation, no such dampening was evident in the present study. The observed red shift in the SPR band can be attributed to morphological evolution of the nanoparticles, suggesting a transition from spherical structures to branched or nanotubular architectures. Furthermore, previous studies, including those based on Mie theory, have demonstrated that an increase in copper content in Au–Cu alloys can also lead to a red shift in the SPR band,^31^ which is consistent with the present findings.

**Fig. 2:**
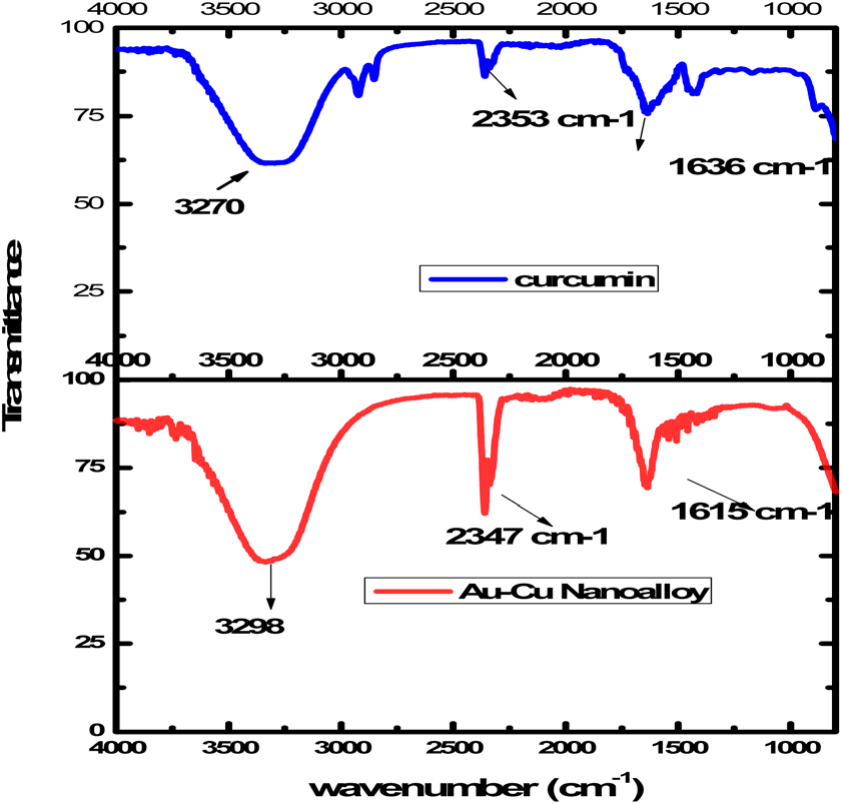
FTIR spectra of curcumin and Au–Cu nanoalloy.

Fourier-transform infrared (FTIR) spectroscopy confirmed the successful functionalization of curcumin on the surface of the Au–Cu nanoalloys. In the FTIR spectrum of Au–Cu NAs (Fig. 2), characteristic absorption bands were observed at 1615 cm^−1^ and 2347 cm^−1^, corresponding to C=O stretching vibrations of the keto-enol form of curcumin and symmetric CH_3_ bending vibrations, respectively. A broad band centred at 3298 cm^−1^ was attributed to O–H stretching vibrations. These spectral features clearly indicate the presence of curcumin moieties on the nanoalloy surface, confirming its dual role as both a reducing and stabilizing agent during synthesis. Furthermore, a slight shift of the characteristic bands toward lower wavenumbers was observed, which can be attributed to interactions between curcumin functional groups and the Au–Cu nanoalloy surface. This shift provides additional evidence of successful surface modification and coordination of curcumin with the bimetallic system.

**Fig. 3:**
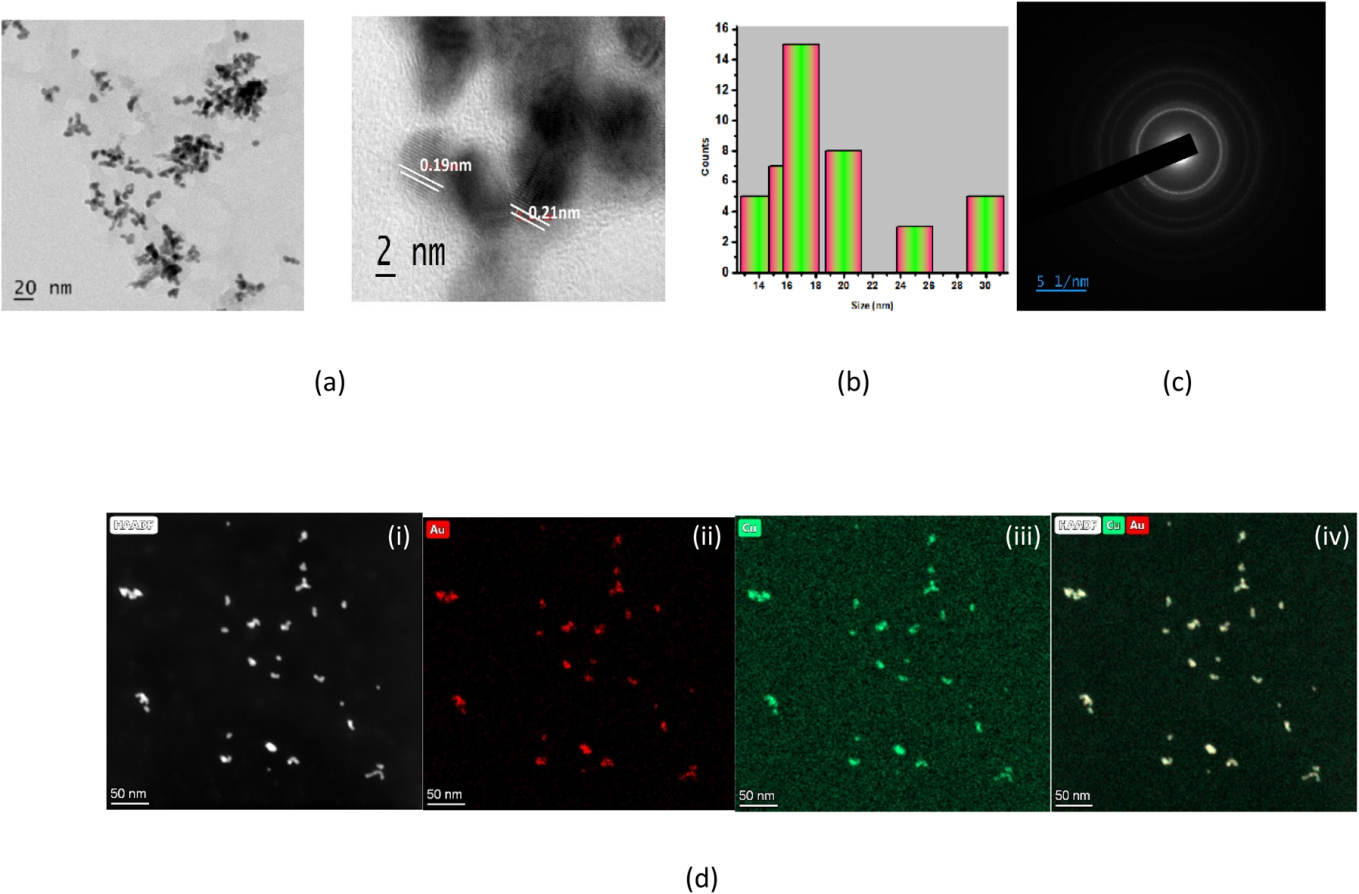
(a) TEM images of Au–Cu NAs under different magnifications. (b) Histogram showing the particle size distribution. (c) SAED pattern. (d) STEM-EDS elemental mapping of Au–Cu NAs – (i) HAADF image, (ii) Au map, (iii) Cu map, (iv) both Au and Cu map.

Dynamic light scattering (DLS) analysis revealed that the synthesized Au–Cu nanoalloys exhibited an average hydrodynamic diameter of approximately 17 nm and a zeta potential of −27.9 mV. The relatively high negative zeta potential indicates excellent colloidal stability, which can be attributed to strong electrostatic repulsion between the nanoparticles. High-resolution transmission electron microscopy (HRTEM, Fig. 3a) images showed that the Au–Cu nanoalloys predominantly exhibit a tubular morphology with well-defined and uniform structural features. The average core particle size was estimated to be approximately 7 nm, which is smaller than the hydrodynamic diameter obtained from DLS measurements due to the contribution of the solvation layer and surface-bound curcumin molecules.

**Fig. 4:**
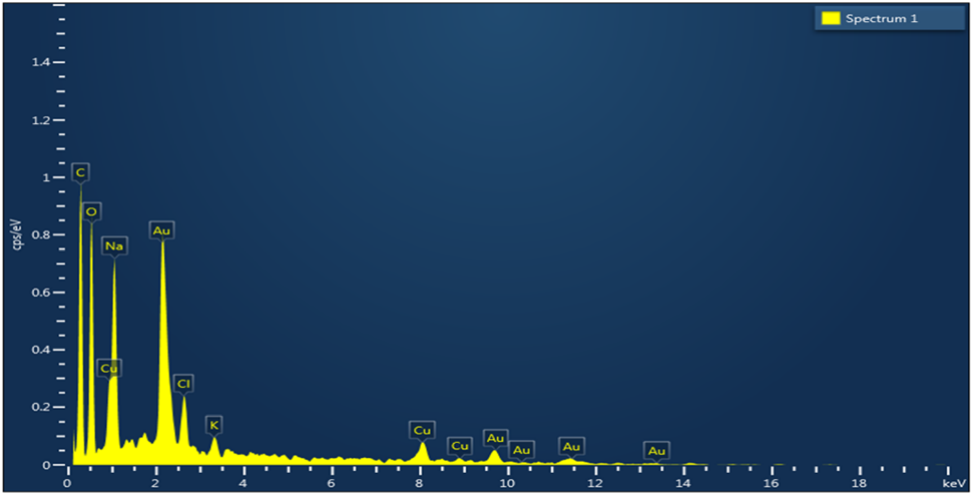
EDX analysis of Au–Cu nanoalloy.

Fig. 3b shows the histogram of the size distribution. HRTEM analysis reveals the presence of nano fringes of 0.19 nm to 0.21 nm on the Au–Cu nanoalloy surface. The lattice fringe spacing of 0.21 nm corresponds to the *d*-spacing of the (111) FCC planes of Au–Cu. The selected area electron diffraction (SAED) pattern shows the crystalline nature of Au–Cu NA. The composition of the nanoalloy was studied through STEM, followed by EDX mapping, which shows that Au and Cu are distributed homogeneously through the branched structure. The chemistry of Au–Cu NA was investigated using HAADF-STEM-EDS and the average composition was found to be 80% Au and 20% Cu. Fig. 3d shows the elemental mapping (STEM-EDS spectrum is given in SI Fig. S2). Scanning electron microscopy (SEM) images (SI Fig. S3) further confirmed the presence of uniformly distributed nanostructures. Energy-dispersive X-ray (EDX) analysis (Fig. 4) verified the presence and homogeneous distribution of Au and Cu elements throughout the nanoalloy surface, confirming successful alloy formation.

### Peroxidase mimicking property of gold nanoparticle

The catalytic efficiency of the Au–Cu nanoalloys was evaluated using hydrogen peroxide (H_2_O_2_) and 3,3′,5,5′-tetramethylbenzidine (TMB) as model substrates. The oxidation of H_2_O_2_ was monitored spectroscopically *via* the conversion of TMB to its oxidized form (TMB^+^) in an acetate buffer. TMB is a widely used chromogenic substrate in peroxidase assays; it is colourless in its reduced state, whereas its oxidized form produces a blue diamine charge-transfer complex. The catalytic activity of the nanoalloys was systematically investigated under different pH conditions, and the highest activity was observed at pH 3.5 (SI, Fig. S4a). Accordingly, this pH was selected as the optimal condition for subsequent catalytic studies. The stability of the Au–Cu nanoalloys under acidic conditions was further confirmed by UV-visible spectroscopy, which showed no evidence of aggregation or peak shifts (SI, Fig. S4b), indicating excellent structural stability.

Reduced TMB exhibits an absorption maximum at 285 nm, whereas oxidized TMB (TMB^+^) shows characteristic absorption peaks at 370, 450, and 652 nm. The progressive increase in absorbance at these wavelengths corresponds to the formation of TMB^+^, driven by the catalytic generation of reactive ˙OH species. The absorbance initially increases linearly with time, followed by a gradual decrease in rate until reaching a plateau, indicating the completion of TMB oxidation.

In this study, the absorbance at 652 nm was monitored to quantitatively track the formation of TMB^+^.^32–36^ Reaction kinetics were recorded using a Thermo Scientific UV-visible spectrophotometer operated in time-scan mode.^37–40^ In the absence of the Au–Cu nanoalloy, no oxidation of TMB was observed, even in the presence of H_2_O_2_, confirming that the reaction does not proceed spontaneously and requires a catalyst, analogous to the behaviour of horseradish peroxidase (HRP). To further validate the catalytic role of the nanoalloy, a control experiment was performed using isolated curcumin (without the nanoalloy) under identical conditions with TMB and H_2_O_2_. No observable colour change occurred, indicating that curcumin alone does not contribute to the catalytic process and that the activity originates exclusively from the Au–Cu nanoalloy.

**Fig. 5:**
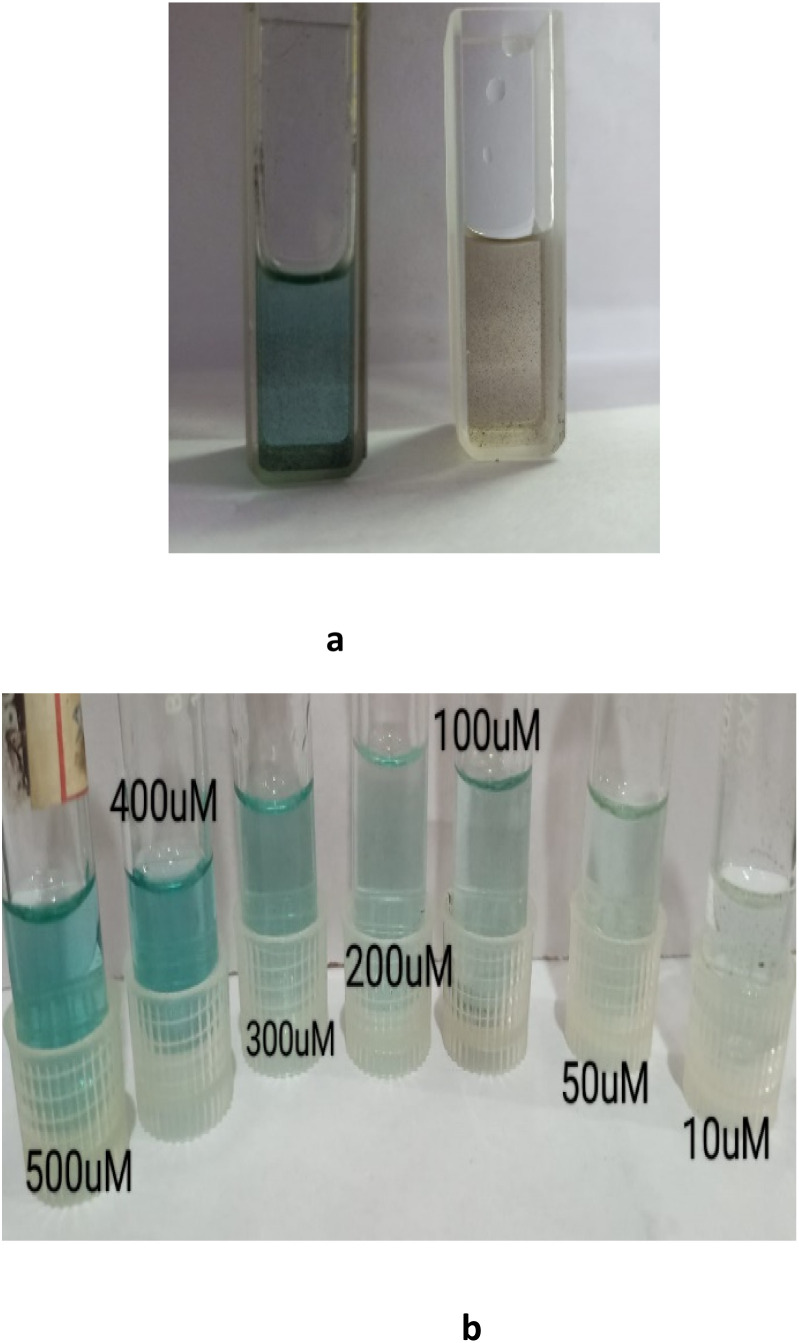
(a) Colour change observed for catalytic performance of the Au–Cu nanoalloy in the presence and in the absence of H_2_O_2_. (b) Colour change for various concentrations of H_2_O_2_.

Fig. 5a illustrates the distinct colour change induced by the nanoalloy in the presence and absence of H_2_O_2_, while Fig. 5b shows the corresponding colorimetric response at varying H_2_O_2_ concentrations ranging from 10 to 500 µM. The detection limit of the proposed method was determined to be 8 µM based on the calibration plot of absorbance *versus* H_2_O_2_ concentration (Fig. S5).

To evaluate the catalytic performance of the Au–Cu nanoalloys, kinetic studies were carried out by varying the concentration of either H_2_O_2_ or TMB while maintaining a constant nanoalloy concentration, following the initial-rate method. Time-dependent changes in the absorbance at 652 nm were monitored to assess the peroxidase-like catalytic activity. The catalytic process can be described by a typical enzymatic reaction mechanism:

1 *E* + *S* ⇌ *ES* → *P* + *E*

where *E* represents the enzyme mimic (Au–Cu nanoalloys), *S* denotes the substrate (TMB or H_2_O_2_), and *P* is the product. The kinetic behaviour of the system follows the Michaelis–Menten model:2
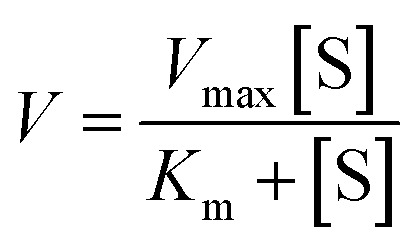
where *V* is the reaction rate, *V*_max_ is the maximum reaction velocity, [S] is the substrate concentration, and *K*_m_ is the Michaelis–Menten constant. To determine the kinetic parameters of the Au–Cu nanoalloy-based peroxidase mimic, the Michaelis–Menten model was applied using the experimentally obtained initial rates. The peroxidase assay was performed using a reaction mixture containing 500 µL of the Au–Cu nanoalloy dispersion, 500 µL of 1 mM TMB, 200 µL of acetate buffer, and 500 µL of H_2_O_2_. Changes in absorbance were recorded using a UV-visible spectrophotometer.

Steady-state kinetic analyses were performed by independently varying the concentrations of H_2_O_2_ and TMB. For H_2_O_2_-dependent kinetics, the concentration of H_2_O_2_ was varied from 0.5% to 4% while maintaining the TMB concentration at 1 mM. For TMB-dependent kinetics, the TMB concentration was varied from 0.1 to 0.5 mM while keeping the H_2_O_2_ concentration constant at 0.3%. The total reaction volume was fixed at 2 mL for all experiments. The concentration of oxidized TMB (TMB^+^) formed over time was determined from time-scan absorbance data using the Beer–Lambert law, with a molar absorption coefficient (*ε*) of 39 000 M^−1^ cm^−1^. The reaction velocity (*V*) was calculated from the slope of the concentration–time profiles. Plots of reaction velocity *versus* substrate concentration yielded typical Michaelis–Menten curves, while double-reciprocal plots (1/*V versus* 1/[S]) produced Lineweaver–Burk plots for kinetic parameter determination.3
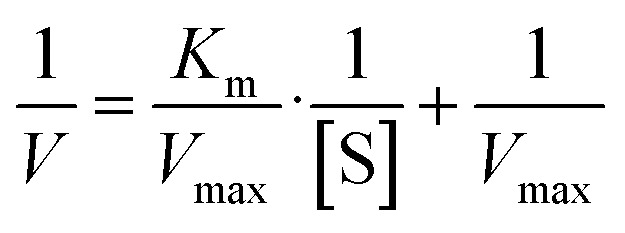


The kinetic parameters *V*_max_ and *K*_m_ were extracted from the slope and intercept of the Lineweaver–Burk plots. Based on the experimental observations, a synergistic catalytic mechanism is proposed for the Au–Cu nanoalloy system. The catalytic process is initiated by the adsorption of H_2_O_2_ molecules onto the nanoalloy surface, followed by their activation and decomposition into reactive oxygen species (˙OH radicals), which subsequently oxidize TMB to TMB^+^. In natural peroxidase enzymes, the catalytic pathway typically follows a ping–pong mechanism, wherein two redox substrates bind sequentially, and the first product is released before the second substrate binds.^30,32^ In contrast, nanozyme systems may follow distinct catalytic pathways depending on their composition. Transition-metal-based nanozymes (*e.g.*, Fe-, Co-, and Cu-based systems) generally operate through valence-state changes arising from redox interactions with substrates, often resembling Fenton-type mechanisms. On the other hand, noble-metal-based nanomaterials exhibit catalytic activity that is largely governed by surface plasmon resonance (SPR)-induced electron transfer. In the present Au–Cu nanoalloy system, the enhanced catalytic performance can be attributed to the synergistic interplay between Cu-mediated redox activity and Au-induced plasmonic electron transfer, leading to improved generation of reactive species and efficient substrate oxidation.

**Fig. 6:**
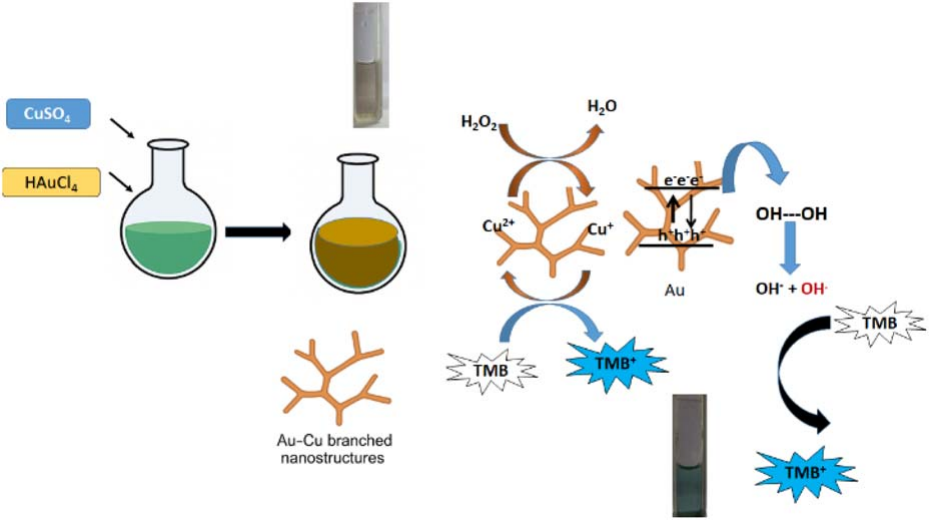
Schematic of the catalytic performance of the Au–Cu nanoalloy.

For transition-metal-based nanozymes, surface metal ions can directly catalyze the decomposition of H_2_O_2_ into hydroxyl radicals (˙OH), which subsequently oxidize the chromogenic substrate. In contrast, noble-metal nanozymes typically exhibit peroxidase-like activity through the adsorption of H_2_O_2_ onto the surface, followed by the O–O bond cleavage, generating ˙OH radicals that oxidize TMB into its coloured product. In the present Au–Cu nanoalloy system, Cu sites play a crucial role in facilitating ˙OH generation *via* a Fenton-like mechanism. The proposed catalytic pathway is illustrated in Fig. 6 (radical scavenging assay results are provided in SI, Fig. S6). The presence of Cu enhances redox cycling, promoting efficient decomposition of H_2_O_2_ into reactive oxygen species.

All experiments were conducted under daylight conditions. As the surface plasmon resonance (SPR) band of the Au–Cu nanoalloy is centred at approximately 550 nm, natural light irradiation can induce plasmon excitation. Upon SPR excitation, hot electrons and holes are generated on the nanoalloy surface. Due to favourable energy alignment, these excited electrons are transferred to the molecular orbitals of adsorbed H_2_O_2_, facilitating its activation to a reactive transition state.^32–34^ This process leads to the cleavage of H_2_O_2_ into hydroxyl radicals (˙OH) and hydroxide ions (OH^−^). Under acidic conditions, the generated OH^−^ ions readily react with H^+^ to form water, which suppresses rapid electron–hole recombination. This inhibition enhances charge separation efficiency, promotes sustained ˙OH radical generation, and ultimately accelerates the overall catalytic reaction rate. Overall, the enhanced peroxidase-like activity of the Au–Cu nanoalloy can be attributed to the synergistic interplay between Cu-mediated Fenton-like redox processes and Au-driven SPR-induced electron transfer, resulting in efficient reactive oxygen species generation and improved catalytic performance.

In this study, we compared the catalytic performance of curcumin-stabilized gold nanoparticles (Cur-GNP), previously reported by our group, with that of the Au–Cu nanoalloy. Based on the calculated Michaelis–Menten constants (*K*_m_) and maximum reaction velocity (*V*_max_), the Au–Cu nanoalloy exhibits superior catalytic activity compared to Cur-GNP. This enhanced performance can be attributed to the synergistic dual mechanistic contributions arising from both transition-metal (Cu-mediated) and noble-metal (Au-mediated) catalytic pathways within the alloyed structure. Fig. 6 illustrates the schematic representation of the catalytic mechanism of Au–Cu nanoalloys. The incorporation of Cu into the Au lattice induces electron donation and compressive lattice strain, resulting in narrowing of the Au 5d band and an upward shift of the d-band centre toward the Fermi level. This electronic modulation enhances adsorbate-metal orbital overlap, strengthens substrate binding, and lowers the activation energy for redox reactions, thereby improving catalytic efficiency. Furthermore, the increased surface area, formation of Au–Cu interfacial ensembles, and strong local electronic perturbations induced by lattice strain—particularly at high-curvature regions—collectively facilitate improved H_2_O_2_ adsorption, accelerated O–O bond cleavage, and enhanced electron transfer kinetics. Notably, branched nanoparticles exhibit higher turnover frequencies, indicating that the observed catalytic enhancement arises predominantly from intrinsic geometric and electronic effects rather than particle size alone.

**Fig. 7:**
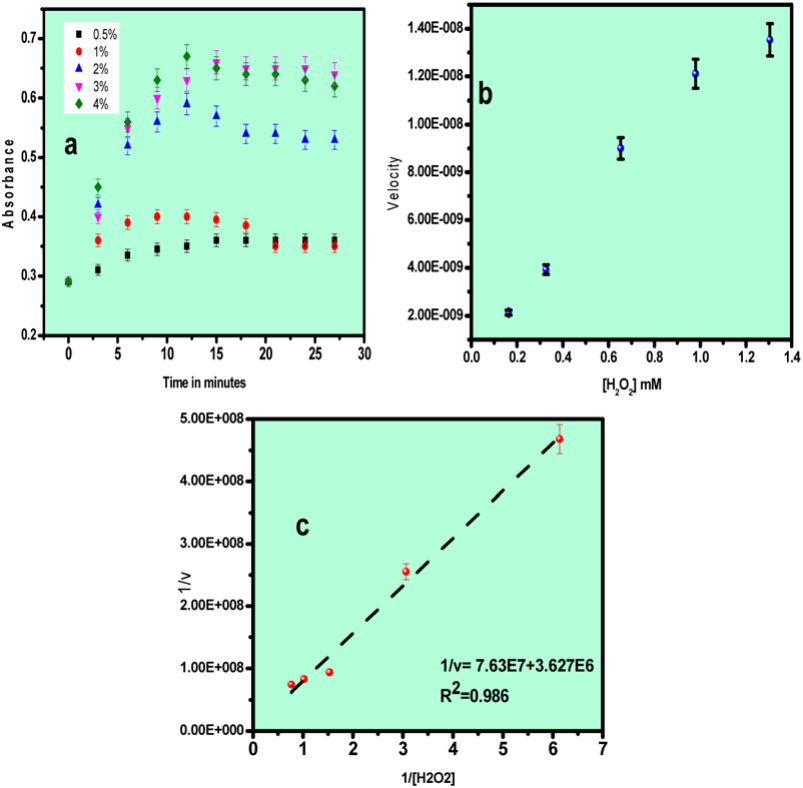
Kinetic plot of the Au–Cu catalyst using different concentrations of H_2_O_2_ as substrate: (a) absorbance *vs.* concentration, (b) Michaelis–Menten plot and (c) Lineweaver–Burk plot.

Fig. 7a shows the plot of absorbance *vs.* time, Fig. 7b corresponds to the Michaelis–Menten plot and Fig. 7c corresponds to the Lineweaver–Burk plot for H_2_O_2_ as substrate, *i.e.*, by keeping TMB concentration as constant and the concentration of H_2_O_2_ was varied from 0.5% to 4%, *V*_max_ = 27.8 × 10^−8^ M s^−1^ and *K*_m_ value was found to be 21.3 × 10^−3^ M.

**Fig. 8:**
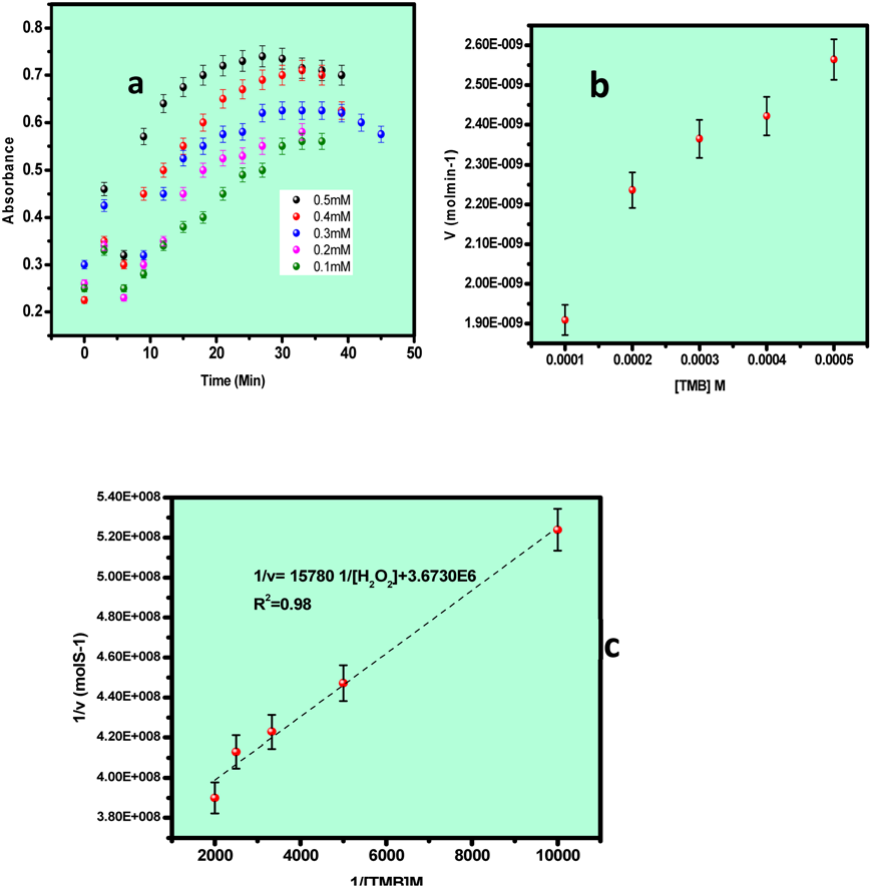
Kinetic plot of the Au–Cu NA catalyst using different concentrations of TMB as the substrate: (a) absorbance *vs.* concentration, (b) Michaelis–Menten plot and (c) Lineweaver–Burk plot.

Fig. 8a presents the absorbance-time plot for the catalytic reaction. Fig. 8b shows the corresponding Michaelis–Menten plot, while Fig. 8c displays the Lineweaver–Burk plot for TMB as the substrate. Using H_2_O_2_ as the substrate and Au–Cu nanoalloy as the peroxidase mimic, the kinetic parameters were determined from the Lineweaver–Burk analysis. The maximum reaction velocity (*V*_max_) was calculated to be 27 × 10^−8^ M s^−1^, and the Michaelis constant (*K*_m_) was found to be 0.0429 × 10^−3^ M.

Lower *K*_m_ values indicate a higher affinity of the catalyst toward its substrate. Based on the obtained kinetic parameters, the *K*_m_ value for TMB is lower for the Au–Cu nanoalloy compared to GNPs, demonstrating that the Au–Cu nanoalloy possesses a stronger binding affinity toward TMB and exhibits higher catalytic efficiency for its oxidation. The *K*_m_ value for H_2_O_2_ is lower for the Au–Cu nanoalloy than for GNPs, indicating a comparatively good affinity toward H_2_O_2_ compared to Cur-GNP, which suggests that the Au–Cu nanoalloy efficiently activates TMB. Table 1 provides a comparative summary of the catalytic activities of various nanostructures, highlighting the performance of the Au–Cu nanoalloy in relation to other reported nanozymes (comparison of detection limits of various gold based nanozymes is given in SI Table S1).

**Table 1:** Comparison of the Michaelis–Menten constant and *V*_max_ of different nano materials

Mimetic material	Substrate	*K*_m_ (10^−3^) M	*V*_m_ (10^−8^) M s^−1^
HRP^34^	TMB	0.433	10
	H_2_O_2_	3.72	8.7
AuNPs/Cit-GNs^40^	TMB	0.0591	14.93
	H_2_O_2_	25.08	21.46
Fe_3_O_4_ (ref. 36)	TMB	0.233	0.176
	H_2_O_2_	479	0.275
Co_3_O_4_ (ref. 36)	TMB	0.103	0.256
	H_2_O_2_	173	0.189
Biogenic GNP^41^	TMB	0.69	
	H_2_O_2_	0.79	
Curcumin stabilised GNP^29^	TMB	0.9	1.98
	H_2_O_2_	4500	7.6
Au–Cu our work	TMB	0.0429	27
	H_2_O_2_	21.20	27.8

### Interference and real sample analysis

**Fig. 9:**
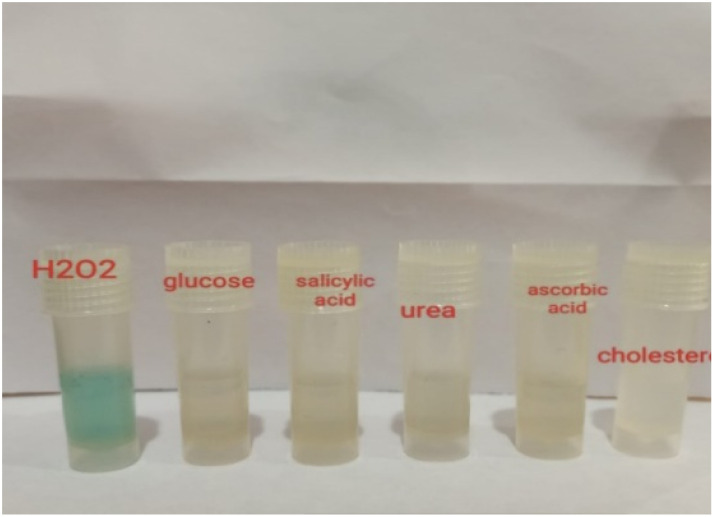
Selectivity study in the presence of interfering species: glucose, salicylic acid, urea, ascorbic acid, and cholesterol.

Interference studies were conducted to evaluate the selectivity of the developed assay. The assay system (in the absence of H_2_O_2_) was tested in the presence of 1 mM concentrations of common potential interferents, including glucose, salicylic acid, urea, cholesterol, and ascorbic acid. The influence of these species on the colorimetric response was examined, and no observable colour change was detected, confirming the high selectivity of the Au–Cu NAs toward H_2_O_2_ (Fig. 9). The limit of detection (LOD) for H_2_O_2_ was determined to be 8 µM, plot of absorbance *vs.* wavelength is given in SI Fig. S6.

To further assess the practical applicability of the nanozyme-based assay, real sample analysis was conducted using commercially available raw milk obtained from a local brand.^42–44^ The milk samples were spiked with known concentrations of H_2_O_2_, and the H_2_O_2_ content was quantified spectrophotometrically. The calculated recovery percentage was approximately 96%, demonstrating the accuracy and reliability of the proposed detection platform (SI Fig. S7).

### Exposition of the electron transfer mechanism *via* electrochemical techniques

**Fig. 10:**
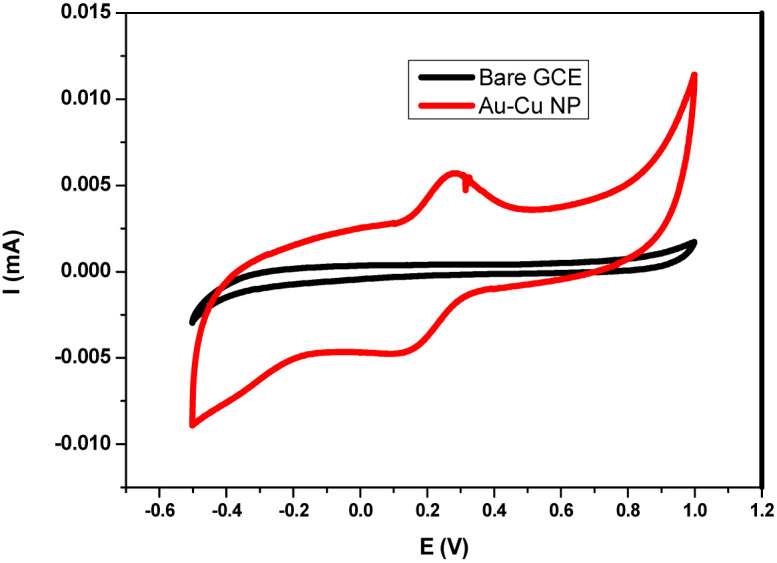
Cyclic voltammetric response of the Au–Cu modified GCE.

To elucidate the electron transfer mechanism, a glassy carbon electrode (GCE) was functionalized with the Au–Cu NAs using polyvinylidene fluoride (PVDF) as a binder for H_2_O_2_ sensing. Cyclic voltammetry (CV) measurements were carried out in an acidic acetate buffer under inert conditions, scanning the potential from +1.2 V to −0.6 V *vs.* calomel electrode. The cyclic voltammogram of the Au–Cu nanoalloy-modified GCE is shown in Fig. 10. A distinct redox peak appears at approximately −0.2 V, corresponding to the Cu^2+^/Cu^+^ redox couple, (comparative CV response of bare GCE, Au–Cu and Cur-GNP is given in SI Fig. S8a). The scan rate variation was recorded and we observed a systematic increase in current with an increase in scan rate (Fig. S8b).

To investigate the electrochemical response toward H_2_O_2_, CVs were recorded at varying concentrations of H_2_O_2_ (SI, Fig. S9). A clear increase in the reduction current was observed near −0.3 V, indicating the catalytic reduction of H_2_O_2_ by the nanoalloy surface. A calibration curve was constructed in the concentration range of 10–80 µM using linear sweep voltammetry (LSV) (SI, Fig. S10). The reduction current increased linearly with increasing H_2_O_2_ concentration, confirming the sensitivity of the system.^29^

The electrochemical process can be described by the following reactions:

4 2Cu^2+^ + 2e^−^ → 2Cu^+^

5 2Cu^+^ + H_2_O_2_ → 2Cu^2+^ + H_2_O + ˙OH

During the cathodic scan, Cu^2+^ on the electrode surface is reduced to Cu^+^. The generated Cu^+^ subsequently reacts with H_2_O_2_, leading to the production of hydroxyl radicals (˙OH), while being oxidized back to Cu^2+^. Thus, with increasing H_2_O_2_ concentration, the regeneration of Cu^2+^/Cu^+^ becomes more pronounced, resulting in an enhanced reduction current.

**Fig. 11:**
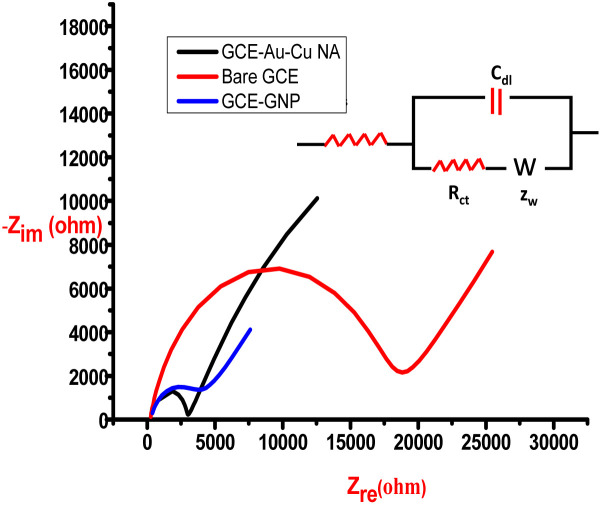
Nyquist plot for GCE, GNP/GCE and Au–Cu NA/GCE in a solution containing equal concentrations of potassium ferrocyanide and potassium ferricyanide, with sodium chloride as the supporting electrolyte. Inset shows the equivalent circuit.

To further understand the electron-transfer characteristics of the nanoalloy, electrochemical impedance spectroscopy (EIS) was conducted. Fig. 11 shows the Nyquist plots for bare GCE, GNP-modified GCE, and the Au–Cu nanoalloy-modified GCE, recorded in a solution containing 1 mM potassium ferricyanide/potassium ferrocyanide with NaCl as the supporting electrolyte. The measured charge-transfer resistance (*R*_ct_) values were Bare GCE: 18 821 Ω cm^2^, GNP-modified GCE: 3891 Ω cm^2^, and Au–Cu nanoalloy-modified GCE: 2887 Ω cm^2^. The significantly lower *R*_ct_ value observed for the Au–Cu nanoalloy electrode indicates faster electron transfer kinetics compared to GNPs. This confirms the superior catalytic efficiency and enhanced electrochemical activity of the Au–Cu nanoalloy. These evidence confirms a dual mechanistic pathway for the catalytic pathway. In our previous work, the catalytic activity of Cur-GNP was mainly attributed to plasmon-induced electron transfer, whereas in the present study, the incorporation of copper introduces an additional redox pathway, leading to enhanced catalytic efficiency.

## Conclusions

In this study, we demonstrated the successful synthesis of Au–Cu branched nanoalloys under aqueous conditions *via* a simple one-pot method using curcumin as both a stabilizing and reducing agent. The resulting nanoalloy exhibited excellent catalytic activity for the detection of hydrogen peroxide (H_2_O_2_) through both colorimetric and electrochemical approaches. The proposed mechanism suggests a synergistic electron-transfer pathway involving plasmon-excited hot carriers and surface redox cycling, collectively contributing to enhanced catalytic performance. Kinetic analysis confirmed Michaelis–Menten behaviour, with favourable *K*_m_ and *V*_max_ values that reflect a strong catalytic affinity of the Au–Cu nanoalloy toward TMB—surpassing, in certain respects, the performance of natural horseradish peroxidase (HRP). Selectivity studies and analyses of real samples revealed negligible interference from common biological molecules. Furthermore, the developed method achieved a low detection limit of 8 µM and demonstrated excellent recovery (∼96%) when applied to raw milk samples. Overall, these findings highlight the potential of the Au–Cu nanoalloy as an effective nanozyme platform for multimodal detection of H_2_O_2_, offering high sensitivity, selectivity, and practical applicability.

## Author contributions

S. P. contributed to writing, conceptualisation, validation, and resources. S. S. carried out all the lab work. P. A. and R. M. helped in electrochemical analysis and interpretation.

## Conflicts of interest

There are no conflicts to declare.

## Supplementary Material

RA-016-D6RA00153J-s001

## Data Availability

The data supporting this article have been included as part of the supplementary information (SI). Supplementary information: the effect of pH, absorbance *vs.* concentration plot, real sample analysis, and scan rate variation plot. See DOI: https://doi.org/10.1039/d6ra00153j.

## References

[cit1] Daniel M.-C., Astruc D. (2004). Chem. Rev..

[cit2] Stratakis M., Garcia H. (2012). Chem. Rev..

[cit3] Saha K., Agasti S. S., Kim C., Li X., Rotello V. M. (2012). Chem. Rev..

[cit4] Zhou W., Gao X., Liu D., Chen X. (2015). Chem. Rev..

[cit5] Hutchings G. J. (2002). Catal. Today.

[cit6] Hashmi A. S. K., Hutchings G. J. (2006). Angew. Chem., Int. Ed..

[cit7] Hutchings G. J. (2009). J. Mater. Chem..

[cit8] Rice K. P., Walker E. J., Stoykovich M. P., Saunders A. E. (2011). J. Phys. Chem. C.

[cit9] Chan G. H., Zhao J., Hicks E. M., Schatz G. C., Van Duyne R. P. (2007). Nano Lett..

[cit10] Zhang H. J., Watanabe T., Okumura M., Haruta M., Toshima N. (2012). Nat. Mater..

[cit11] Zhang L. F., Zhong S. L., Xu A. W. (2013). Angew. Chem., Int. Ed..

[cit12] Gao L. (2007). et al.. Nat. Nanotechnol..

[cit13] Xie J., Zhang X., Wang H., Zheng H., Huang Y. (2012). TrAC, Trends Anal. Chem..

[cit14] Manea F., Houillon F. B., Pasquato L., Scrimin P. (2004). Angew. Chem., Int. Ed..

[cit15] Huang Y., Ren J., Qu X. (2019). Chem. Rev..

[cit16] Ling P. (2020). et al.. ACS Appl. Mater. Interfaces.

[cit17] Liu F., He J., Zeng M., Hao J., Guo Q., Song Y., Wang L. (2016). J. Nanopart. Res..

[cit18] Wang S. (2018). et al.. Anal. Bioanal. Chem..

[cit19] Zhang J., Claverie J., Chaker M., Ma D. (2017). ChemPhysChem.

[cit20] Fang H., Yang J., Wen M., Wu Q. (2018). Adv. Mater..

[cit21] Coviello V., Forrer D., Amendola V. (2022). ChemPhysChem.

[cit22] Meng X. (2024). et al.. Nano Lett..

[cit23] Sytwu K., Vadai M., Dionne J. A. (2019). Adv. Phys.: X.

[cit24] Forsythe R. C., Cox C. P., Wilsey M. K., Müller A. M. (2021). Chem. Rev..

[cit25] Vassalini I. (2017). et al.. Angew. Chem., Int. Ed..

[cit26] Alexander D. T. L. (2019). et al.. Nano Lett..

[cit27] He W., Wu X., Liu J., Hu X., Zhang K., Hou S., Zhou W., Xie S. (2010). Chem. Mater..

[cit28] Kshirsagar P. G., De Matteis V., Pal S., Sangaru S. S. (2023). Nanomaterials.

[cit29] Sudhesh P., Sruthi S., Jose M. (2025). et al.. Sci. Rep..

[cit30] Spataro G. M., Yang J., Coviello V., Agnoli S., Amendola V. (2024). ChemPhysChem.

[cit31] Mi X., Zhang T., Zhang B., Ji M., Kang B., Kang C. (2021). et al.. FrontChem.

[cit32] Jin S., Wu C., Ye Z., Ying Y. (2019). Sens. Actuators, B.

[cit33] Lin Y., Ren J., Qu X. (2014). Adv. Mater..

[cit34] Lou-Franco J., Das B., Elliott C., Cao C. (2020). Nano-Micro Lett..

[cit35] Navyatha B., Singh S., Nara S. (2021). Biosens. Bioelectron..

[cit36] Chen Z. (2012). et al.. ACS Nano.

[cit37] Dong J. (2014). et al.. ACS Appl. Mater. Interfaces.

[cit38] Josephy P. D., Eling T., Mason R. P. (1982). J. Biol. Chem..

[cit39] Gu S., Risse S., Lu Y., Ballauff M. (2020). ChemPhysChem.

[cit40] Drozd M., Pietrzak M., Parzuchowski P. G., Malinowska E. (2016). Anal. Bioanal. Chem..

[cit41] Chen X. (2014). et al.. Dalton Trans..

[cit42] Zhang Y. (2020). et al.. J. Phys. Chem. Lett..

[cit43] Chepkasov I. V., Baidyshev V. S., Kvashnin A. G. (2023). Phys. Rev. B.

[cit44] Cao S., Tao F. F., Tang Y., Li Y., Yu J. (2016). Chem. Soc. Rev..

