# Supplementary material for: Bio-inspired branched Au–Cu nanoalloys for achieving synergistic dual-pathway peroxidase-like activity
Source: RSC Adv. 2026 Apr 24;16(24):21629–38. doi: 10.1039/d6ra00153j (PMC13108399; doi:10.1039/d6ra00153j)
Supplement: RA-016-D6RA00153J-s001 [file RA-016-D6RA00153J-s001.pdf]

Supplementary Information (SI) for RSC Advances.
This journal is © The Royal Society of Chemistry 2026

Supporting Information

Bio-Inspired Branched Au–Cu Nanoalloys as Synergistic Dual-Pathway Peroxidase like activity

S. Priya.[*a], *S. Sruthi* [b], *P. Aiswarya* [c] *and R. Manu* [c]

a-NSS college Ottapalam, b-NSS College Nemmara, c-SVNSS college

Keywords-Au-Cu Nanoalloy, Coreduction, Green synthesis, Peroxidase mimicking, Multimodal sensing

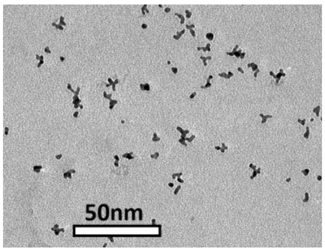

Fig S1a-TEM image after 1 year

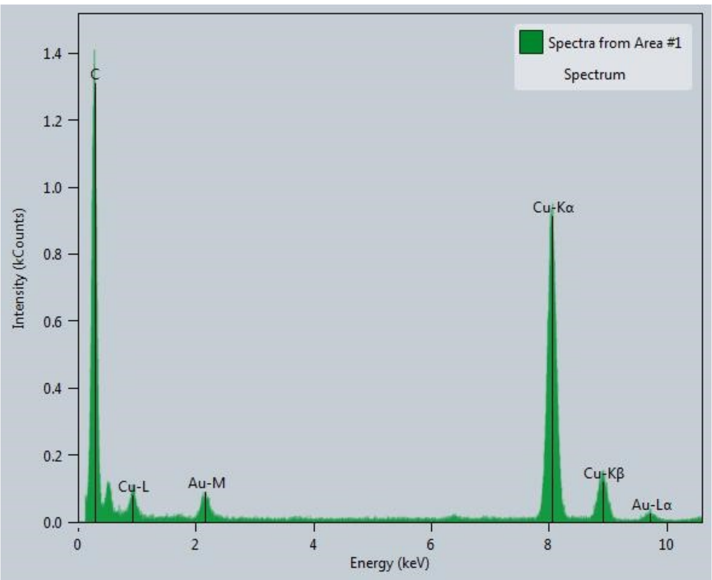

Fig S2 -HAADF EDS spectrum

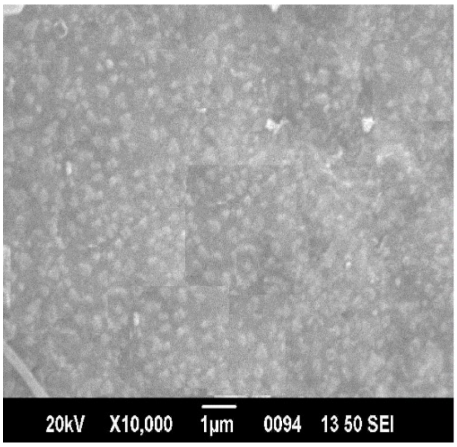

Figure S3-SEM image of Au-Cu NA

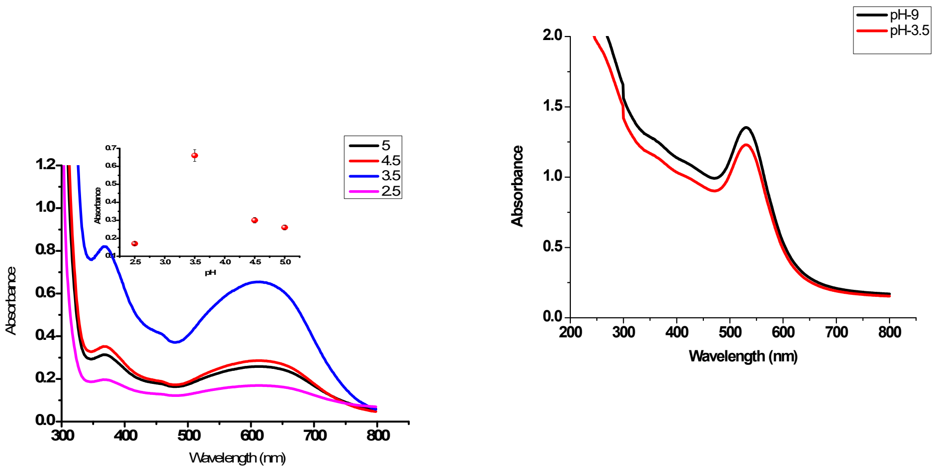

a b

Fig S4 a- Plot of Absorbance for various pH conditions. Inset show the plot of abs vs pH b- UV-Vis spectrum of Au-Cu NA at pH 9 and 3.

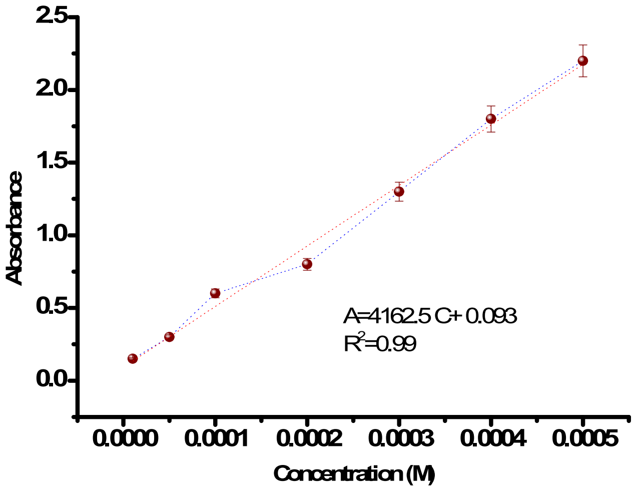

Fig S5- Plot of Abs vs Conc for various concentration of H2O2 from 10μM to 500μM

Radical scavenging assay

The hydrogen peroxide (H_2O_2) scavenging activity of the samples was evaluated according to the method described by Ruch et al. A 40 mM solution of H_2O_2 was prepared in phosphate buffer and mixed with the test samples. The remaining concentration of H_2O_2 was measured spectrophotometrically by recording the absorbance at 230 nm. The percentage of H_2O_2 scavenging activity was then calculated accordingly. A_0 represents the absorbance of the control sample, while A_1 denotes the absorbance after treatment with ascorbic acid, Cur-GNP and Au-Cu NA.

The figures below illustrate the radical scavenging activity of ascorbic acid,and Au-Cu NA. A progressive decrease in absorbance at 230 nm was observed with increasing concentrations of ascorbic acid and Au-CuNA, indicating enhanced H_2O_2 scavenging activity. Scavenging efficiency is found to be higher for Au-Cu NA. This shows that Au-Cu NA has god catalytic efficiency to rupture O-O bond of H_2O_2.

$$\frac{Ao-A1}{Ao} X100$$

(Reference-Keser, Serhat & Celik, Sait & Türkoğlu, Semra & Yilmaz, Okkes & Turkoglu, Ismail. (2012). Hydrogen Peroxide Radical Scavenging and Total Antioxidant Activity of Hawthorn. Chem J. 2.)

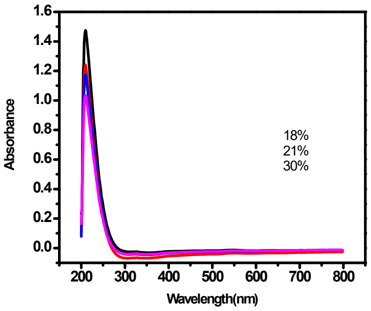

a

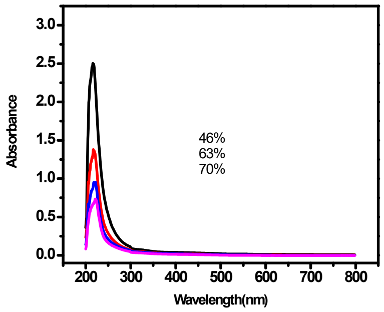

b

Fig S6-Radical scavenging efficiency of a- ascorbic acid b-Au-Cu NA

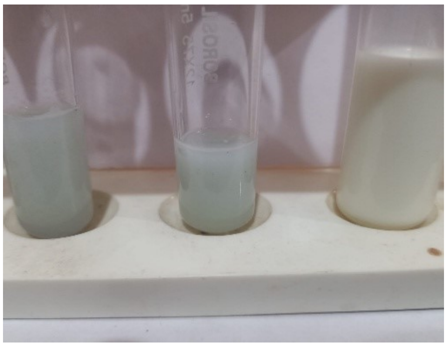

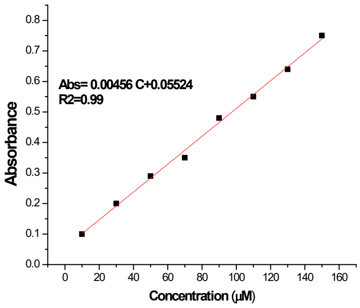

	Concentration Spiked	Recovered concentration	Recovery %
1	90μM	87 μM	96%
2	130 μM	125 μM	95%

Figure-S7 Real sample analysis and colour change observed for various concentrations of H_2O_2

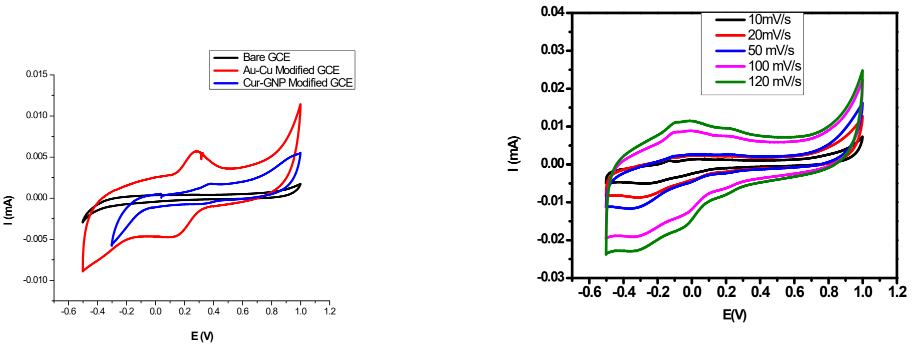

Fig S8 a- Cyclic voltametric response of GCE, Au-Cu nano particle and Gold nanoparticle modified electrode vs SCE. b-Scan rate variation.

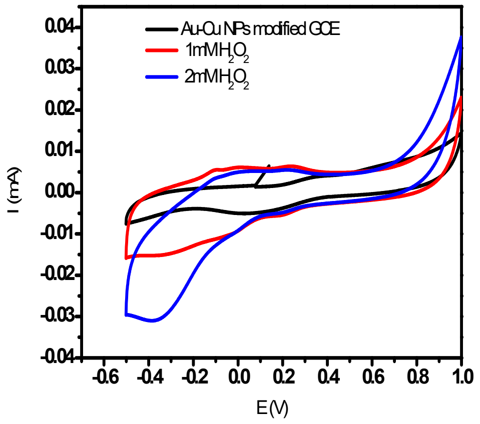

Fig S9- **Cyclic voltametric response of Au-Cu modified electrode in various concentration of H_2O_2.**

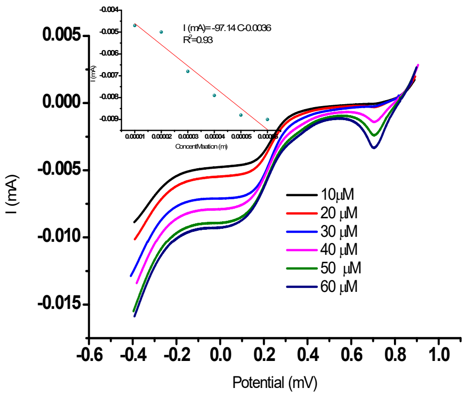

FigS10- Calibration plot for the concentration range from 10-60 µM.

Table S1-Showing comparison of detction limits of various gold based nanozymes

SI No.	Type	Detection limit	Reference
1	Gold nano rattles	0.5µM	1
2	Graphene-oxide gold	1.9 nM	2
3	Au@PtNP/GO	1.6 µM	3
4	Fe3O4@PB@Au	13.4 µM	4
5	Au@CMs	7 µM	5
6	Cu doped carbonized bacterial cellulose (BC) nanofibers	2.2 µM	6
7	gold nanoparticles deposited CeO_2	3 µM	7

References

1.S. Ghosh, P. Singh, S. Roy, K. Bhardwaj and A. Jaiswal, ChemBioChem, 2022, 23,

2. G. H. Jin, E. Ko, M. K. Kim, V.-K. Tran, S. E. Son, Y. Geng, W. Hur and G. H. Seong, Sens. Actuators B Chem., 2018, 274, 201–209

3. E. Ko, V. K. Tran, S. E. Son, W. Hur, H. Choi and G. H. Seong, Sens. Actuators B Chem., 2019, 294, 166–176.

4. S. Ruan, S. Chen, Y. Tu, Y. Liu, R. You, Q. Lin and Y. Lu, Microchem. J., 2024, 205, 111377.

5. Y. Wei, J. Lu, Y. Xu, X. Song, Y. Yu, H. Zhang and X. Luo, Microchem. J., 2023, 193, 109113.

6. S. Zhang, F. Ding, S. Huang and X. Ren, Chem. Eng. J., 2025, 507, 160602.

7. N. Jabiyeva, B. Çakıroğlu and A. Özdemir, J. Photochem. Photobiol. A Chem., 2024, 452, 115576.
